# Editorial: Advances in alternative methods in preclinical pharmacology and toxicology

**DOI:** 10.3389/fphar.2023.1225806

**Published:** 2023-07-19

**Authors:** Andresa Heemann Betti, John Gerry Kenna, Terry R. Van Vleet, Palanisamy Aruselvan, Liz Girardi Müller

**Affiliations:** ^1^ Bioanalysis Laboratory, Institute of Health Sciences, Feevale University, Novo Hamburgo, Brazil; ^2^ Safer Medicines Trust, Kingsbridge, United Kingdom; ^3^ Development Biological Sciences, North Chicago, IL, United States; ^4^ Scigen Research and Innovation Pvt Ltd., Thanjavur, India; ^5^ Graduate Program in Environmental Sciences, Community University of Chapecó Region, Chapecó, Brazil

**Keywords:** preclinical assays, drug discovery, toxicity, *in vitro*, *in vivo*

Historically, animal models have played an unique role in drug development and toxicological research, offering important insights into biological mechanisms that other approaches cannot replicate (Robinson et al., 2019; Gorzalczany and Basso, 2021). However, given the rising ethical concerns and practical limitations associated with animal experimentation, alternative testing methods guided by the 3 Rs principles—reduction, refinement, and replacement—have been proposed and progressively implemented (National Research Council, 2011). Furthermore, another “R” has been proposed, referring to responsibility for the animal, promoting animal welfare through improvements in the experimental animals’ social life, development of advanced scientific methods to objectively determine sentience, consciousness, the experience of pain and intelligence, as well as effective involvement in the professionalization of the public discussion on animal ethics.

Regarding all these concerns, alternative techniques must always be used, when available, instead of animal experimentation. These alternative methods refer to any technique that can be applied to replace, reduce or refine the laboratory use of animals (Pound and Ritskes-Hoitinga, 2020). The development of alternative methods for pharmacological and toxicological research includes, but it is not limited to, the use of invertebrate or lower vertebrates (which are genetically related to the higher vertebrates), the application of *in vitro* cell and tissue cultures, and the use of microorganisms to understand fundamental aspects of cellular biology. While these alternatives typically offer advantages in terms of time, efficiency, and cost-effectiveness, not all proposed methods have been able to realize these benefits.

The alternative methods established for a specific purpose (e.g., pharmacological or toxicological studies) must be relevant and reproducible, and need to be validated by a regulatory agency, in order to confirm the robustness, reliability and translatability (Meigs et al., 2018). This regulamentation is accepted by either national or international regulatory authorities, such as the OECD, INVITTOX, pharmacopeias, ISO (Gorzalczany and Basso, 2021). Upon approval, these methods become obligatory for the toxicological and/or pharmacological assessment of substances and products. Consequently, they attain international recognition and are correctly employed in the scientific sphere (Meigs et al., 2018).

The development and implementation of non-animal strategies is an ongoing process. Once alternative methods are validated, disseminated and firmly anchored by the legislation worldwide, they significantly contribute to reducing animal use. Validated alternative methods, although not fully replacing animal tests, have a substantial impact on lowering the number of animals used, as they can be applied across various fields, such as cosmetics, medicines, and pesticides. While total replacement of animal testing remains the ultimate objective, the complete cessation of animal use in scientific procedures continues to be a formidable challenge. This is mainly due to the complexities associated with generating substitutes that can mirror the depth of knowledge provided by animal research (Combrisson, 2017).

This Research Topic is therefore dedicated to gathering potential novel methods designed to address the inherent difficulties tied to the use of experimental animals in drug development and toxicological tests. Following concerted efforts from the journal, editors, reviewers, and contributors, we successfully published five high-quality articles. Herein, we provide a brief summary of each paper.

This Research Topic includes two review articles. Quintás et al. shed light on the current advancements in metabolomics applied to *in vitro* hepatotoxicity studies, offering a valuable framework that can be extrapolated to other fields where metabolomics approaches are beneficial or required. Xiu et al. highlighted the merits of using *Drosophila melanogaster* (fruit flies) as a potent model to assess the therapeutic potential of phytochemicals against Inflammatory Bowel Disease (IBD). Their review underscores the role of *D. melanogaster* as a screening platform in drug discovery, elaborates on the conserved molecular pathways as therapeutic targets for IBD between mammals and flies, probes the feasibility of the *Drosophila* model in IBD research, and summarizes the natural products identified for IBD treatment using this approach.

Futosi et al. developed an innovative assay system for the flow cytometric analysis of intracellular tyrosine phosphorylation in circulating mouse leukocytes. This study represents an important step forward, especially considering the pivotal transition of developing tyrosine kinase inhibitors from an *in vitro* to an *in vivo* phase.

Faramarzi et al. presented computational models for predicting blood-brain barrier (BBB) permeability. They designed statistical-based quantitative structure-activity relationship (QSAR) models to estimate BBB permeability of drugs predicated on their chemical structures.

Zebrafish (*Danio rerio*)*,* as lower vertebrates, are commonly employed in studies regarding brain functions, neurological diseases, and drug toxicity (Strähle et al., 2012). Expanding on this, Shin et al. proposed an innovative method for the pharmacological evaluation of antiepileptic drugs using electroencephalogram signals (EEG) from adult zebrafish. This approach mirrors the method used to evaluate the antiepileptic effects of drugs in mammals, thereby suggesting that their proposed method could expedite drug development cycles and decrease costs.

In conclusion, this Research Topic has provided novel experimental data and insightful reviews that enrich our understanding of alternative methods in preclinical pharmacology and toxicology. We are hopeful that these contributions will continue to drive this field, especially regarding the use of animals only when alternative methods are not available for the purpose. In this process, all researchers must go beyond what is legally required, developing a culture of care to improve animal welfare, scientific quality, care of staff and transparency for stakeholders, when animals have to be used (Lewis, 2019). So far, much progress has been made on reducing animal experimentation; however, there is a need for greater awareness of alternatives to animal experiments among scientists and easier access to advanced modeling technologies (Kiani et al., 2022). Alternative methods seek to ensure the rational and respectful use of laboratory animals and maintain an adequate projection in terms of bioethical considerations (Gorzalczany and Basso, 2021). The complete elimination of all unnecessary animal experimentation requires further studies on the development of reliable methodologies in biomedical research.
